# Endovascular Treatment of Large Unruptured Fusiform Fenestrated Vertebrobasilar Junction Aneurysm

**DOI:** 10.7759/cureus.1219

**Published:** 2017-05-03

**Authors:** Saeed A. Alqahtani, Daniel R Felbaum, Alex Tai, Ai-Hsi Liu, Rocco A Armonda

**Affiliations:** 1 Neurosurgery, Medstar Georgetown University Hospital; 2 Neurointerventional Radiology, Medstar Washington Hospital Center

**Keywords:** basilar artery fenestration, vertebral artery, endovascular treatment of aneurysm, fusiform aneurysm, coil embolization, stent-assisted coiling, unruptured intracranial aneurysm

## Abstract

Fenestrated vertebrobasilar junction aneurysms are rare vascular lesions. Microsurgical intervention is extremely difficult due to the complex anatomy in the vicinity of these aneurysms. Endovascular neurosurgery appears to be safe and should be considered as the first modality of treatment. This case study details the treatment of an unruptured fusiform fenestrated vertebrobasilar junction aneurysm with endovascular occlusion with stent-assisted coiling. The optimal angiographic exposure and selective microcatheterization of the aneurysm were challenging due to the patient’s body habitus, and the aneurysm was large with one dominant fenestrated limb.

## Introduction

Vertebrobasilar junction aneurysms are very rare, complex lesions and are mostly associated with basilar artery fenestration [1-3]. The microsurgery approach of such aneurysms is difficult due to both a lack of optimal surgical exposure and the complex vicinity of the brainstem anatomy. An endovascular neurosurgical approach becomes the modality of choice in treating these aneurysms [4-5]. We report a unique case of a large unruptured fusiform fenestrated vertebrobasilar junction aneurysm treated with endovascular occlusion of the aneurysm with stent-assisted coiling through a dominant limb of the fenestration without encountering complications.  

## Case presentation

History and examination

**Figure 1:**
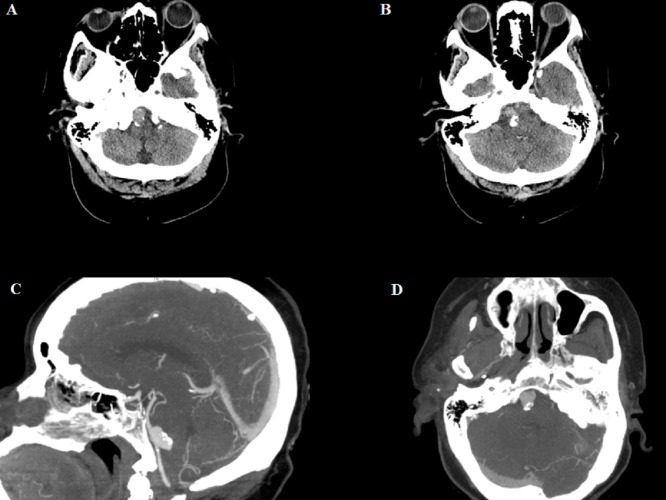
Non-contrast CT scan of the head and CTA of the head A, B) Axial non-contrast-enhanced head CT scan showing a peripherally calcified extra-axial soft tissue density structure along the right anterior aspect of the medulla. C, D) Sagittal and axial CTA of the head illustrating a large, saccular, and irregularly shaped aneurysm. CT: computed tomography; CTA: computed tomography angiography

A 65-year-old right-handed, morbidly obese African-American woman with a past medical history of hypertension and hepatitis C presented to our hospital after experiencing a fall while walking. During her fall, the left side of her face hit the ground. She denied any loss of consciousness. On physical examination, we found no neurological focal deficit, and her vital signs were stable. A computed tomography (CT) scan of her head did not show any intracranial hemorrhage; however, it showed a 1.7-cm × 1.6-cm × 1.2-cm hyperdense lesion with coarse rim calcification anterior to the medulla (Figure 1A, 1B). A computed tomography angiography showed a large, unruptured aneurysm at the junction of the left vertebrobasilar artery (Figure 1C, 1D).

**Figure 2:**
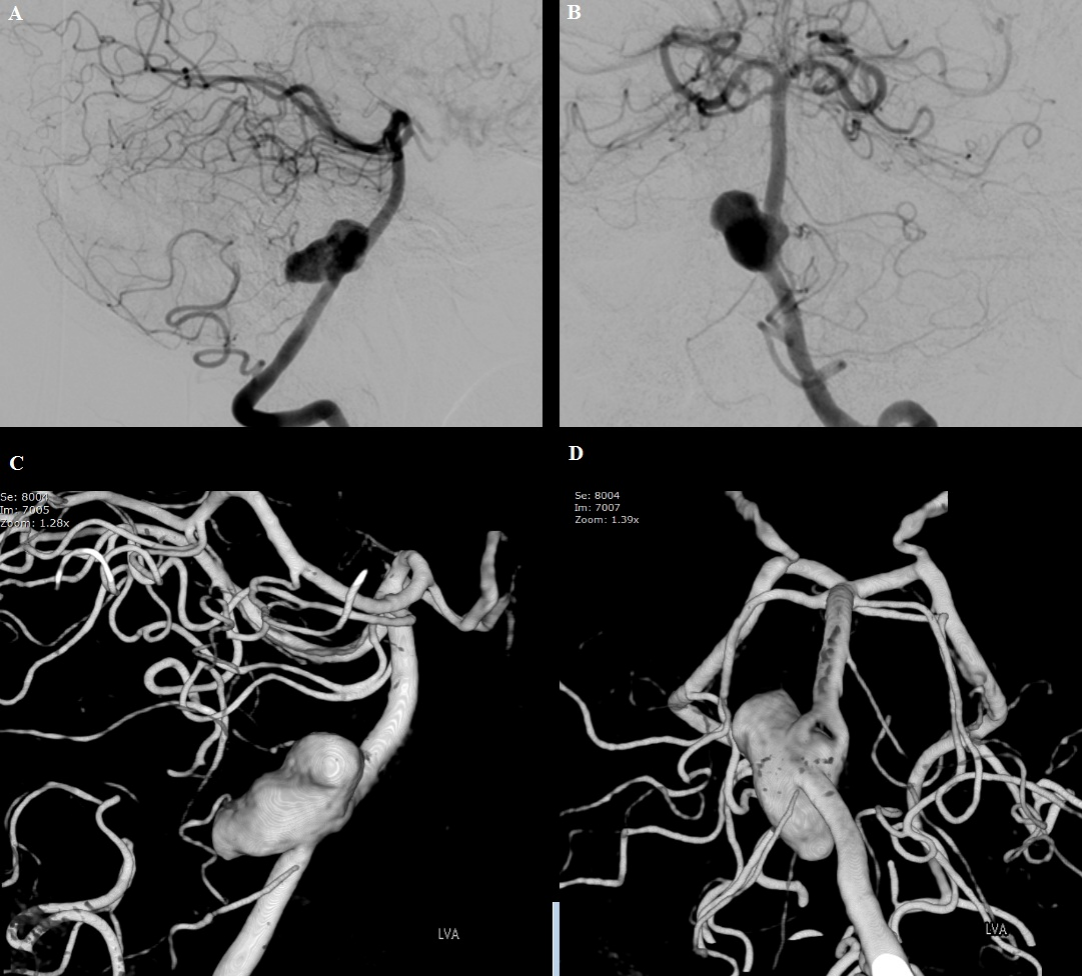
Left vertebral artery angiography with 3D RA A, B) Lateral and anteroposterior arterial phase angiography of the left vertebral artery showing a 20.5-mm × 9.5-mm × 9.1-mm aneurysm arising from the dorsal aspect of the vertebrobasilar junction. C, D) 3D RA with dual-volume reconstruction illustrating a fenestration at the proximal basilar artery just beyond the left vertebrobasilar junction. 3D: three-dimensional; RA: rotational angiography

Subsequent digital subtraction angiography and three-dimensional rotational angiography with dual-volume reconstruction demonstrated a fenestration at the proximal basilar artery just beyond the left vertebrobasilar junction. An aneurysm measuring 20.5 mm × 9.5 mm × 9.1 mm arose from the dorsal aspect of the vertebrobasilar junction, and it incorporated the proximal end of the fenestration as shown in Figure 2A-2D.

The left limb of the fenestration, measuring 2.3 mm × 2.7 mm, was the dominant limb. The proximal basilar artery measured 3.7 mm, and the intradural left vertebral artery measured 3.3 mm. The right vertebral artery was hypoplastic and ended in a posterior inferior cerebellar artery (PICA). The patient was discharged home with a scheduled follow-up assessment in the neurosurgery clinic. The decision was made to treat her brain aneurysm using an endovascular approach. An intraluminal support device (i.e., stent) would be needed to ensure patency of at least one limb of the fenestration. The patient started on dual antiplatelets (clopidogrel 75 mg daily and aspirin 325 mg daily) in preparation for the endovascular surgery.

Endovascular approach

**Figure 3:**
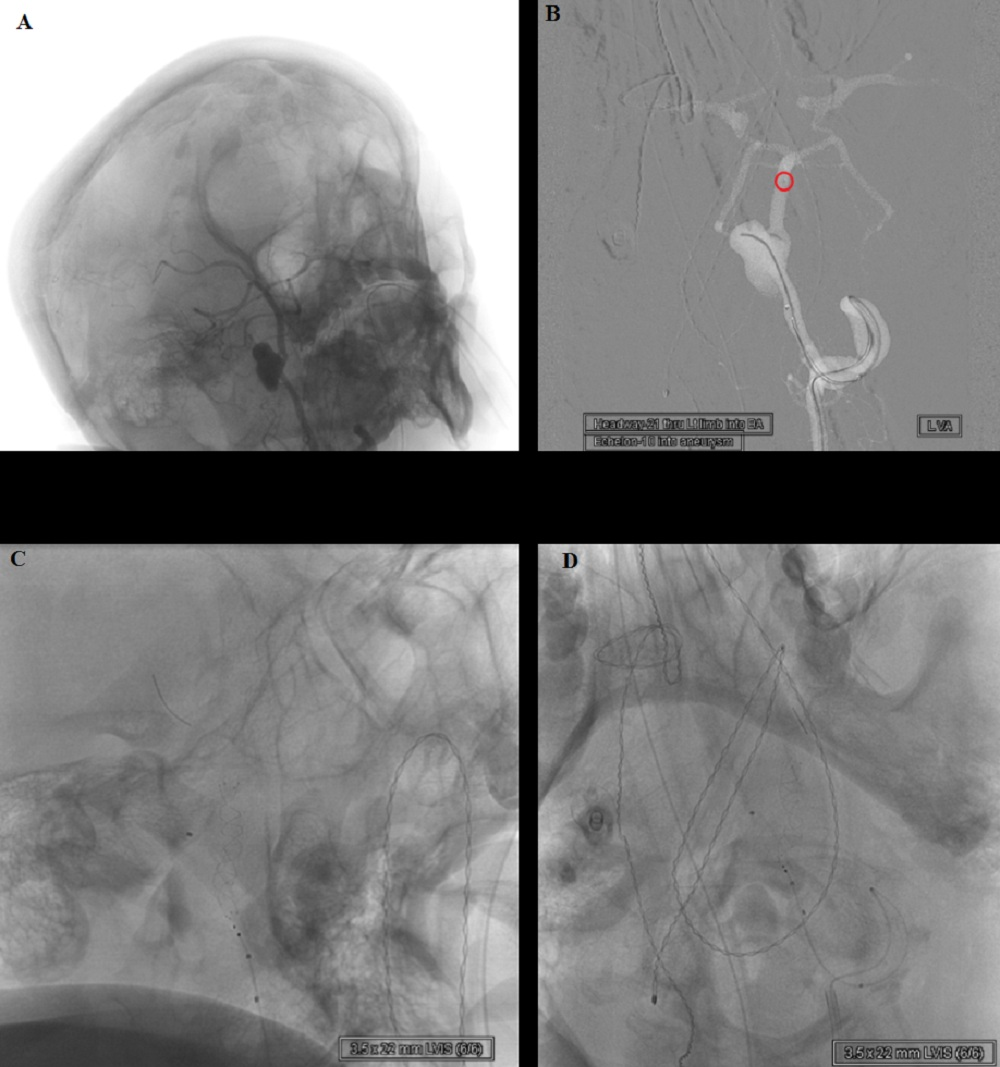
Head positioning and left vertebral artery view A) Head radiograph showing neck extension and head positioning for optimal angiographic exposure of the aneurysm. B) Left vertebral artery anteroposterior angiography road map view showing the tip of the microcatheter at the basilar artery tip (red circle) along with microcatheter micro guidewire combination at the aneurysm sac. C, D) Lateral and anteroposterior radiography projection of the left vertebral artery illustrating the 3.5-mm × 22-mm LVIS device fully deployed from the midbasilar arterial trunk towards the distal intradural left vertebral artery, across the neck of the aneurysm. LVIS: low-profile visualized intraluminal support

The patient was electively admitted to our hospital for the endovascular surgery. To obtain optimal angiographic exposure of the aneurysm, the patient's neck was extended and turned towards the right side (Figure 3A). Under general anesthesia, the right femoral artery was punctured by a 21-gauge micropuncture needle, and a 10-cm 6 French sheath (Terumo, NJ, USA) was inserted. A 4 French H1 catheter (Cordis, Miami Lakes, FL) and a 0.035-inch Glidewire® hydrophilic coated guidewire (Terumo, NJ, USA) were used to catheterize the left vertebral artery. The 4 French H1 catheter in the high cervical left vertebral artery was removed over a 260-cm Newton guidewire (Cook, IN, USA). Then, a 6 French right groin arterial sheath was also removed and exchanged for a 6 French, 80-cm shuttle sheath (Cook, IN, USA). The tip of the shuttle sheath was placed adjacent to the origin of the left subclavian artery. Then, a 6 French 95-cm Chaperon guiding catheter (MicroVention-Terumo, Inc., Tustin, CA) was inserted into the shuttle sheath. Using the Glidewire, the cervical left vertebral artery was selected, and the tip of the chaperone guiding catheter was advanced to the high cervical left vertebral artery. The shuttle sheath was also advanced into the midportion of the cervical left vertebral artery for proximal support.

A combination of a Headway-21 microcatheter (156 cm) (MicroVention-Terumo, Inc., Tustin, CA) and a Transcend-14 Platinum (Boston Scientific, Marlborough, MA) micro guidewire was introduced into the guiding catheter. We encountered some difficulty to select the dominant left limb of the fenestration. The micro guidewire was exchanged to a Synchro-14 Standard (Stryker Neurovascular, Fremont, CA) micro guidewire. Using the new micro guidewire, we were able to select the dominant left limb of the fenestration and then advance the tip of the Headway-21 microcatheter towards the basilar apex. A combination of an Echelon-10 microcatheter (ev3, Irvine, CA) and the Synchro-14 micro guidewire was then introduced into the Chaperone guiding catheter alongside the Headway microcatheter. The lumen of the aneurysm was catheterized by the Echelon microcatheter micro guidewire combination (Figure 3B).

A 3.5-mm × 22-mm low-profile visualized intraluminal support (LVIS) device (MicroVention-Terumo, Inc., Tustin, CA) was introduced into the Headway-21 microcatheter. The LVIS device was deployed from the midbasilar arterial trunk towards the distal intradural left vertebral artery, across the neck of the aneurysm as shown in Video 1 and Figure 3C, 3D. A follow-up angiogram obtained through the guiding catheter in the left vertebral artery demonstrated patency of the LVIS device from the midbasilar arterial trunk towards the distal intradural left vertebral artery. The Headway-21 microcatheter and the LVIS device delivery wire were removed.

**Video 1:** Deployment of LVIS Intraluminal Support Device LVIS intraluminal support device deployment from the mid basilar arterial trunk towards the distal intradural left vertebral artery, across the neck of the aneurysm. LVIS: low-profile visualized intraluminal support

**Figure 4:**
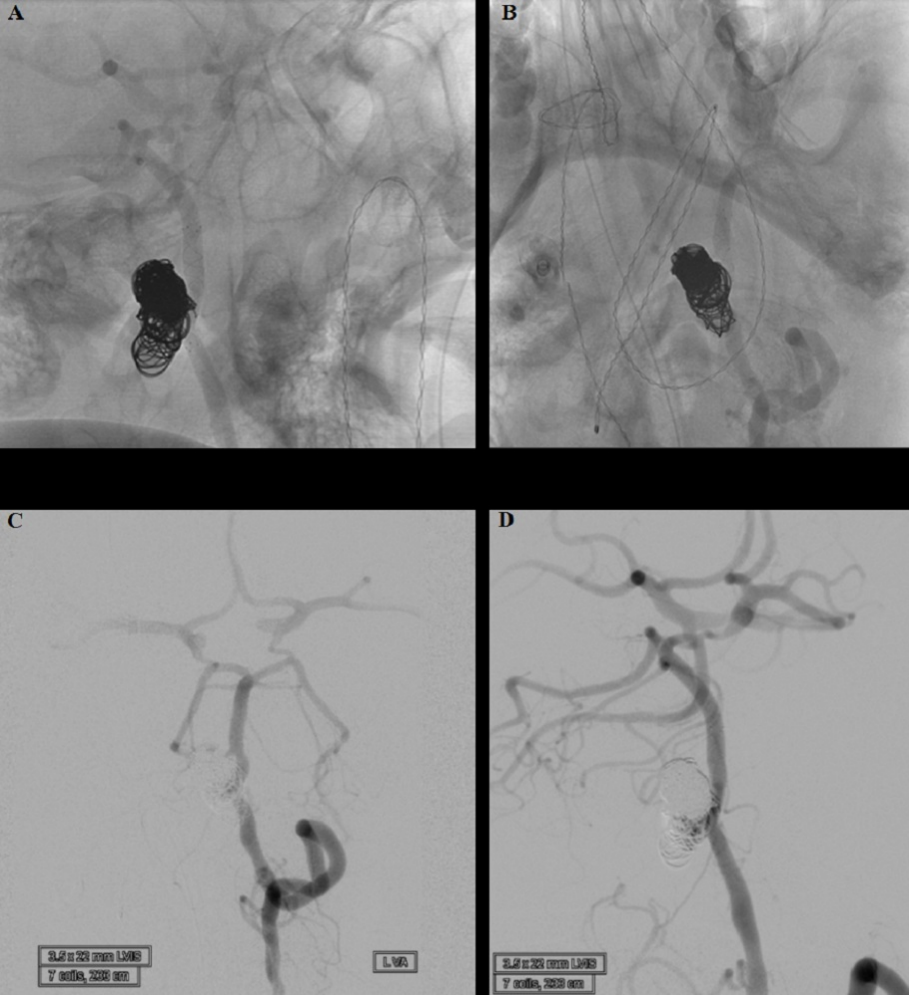
Left vertebral artery after coiling A, B) Subtracted view of the lateral and anteroposterior arterial phase angiography of the left vertebral artery showing successful stent-assisted coiling embolization of the aneurysm. C, D) Anteroposterior and lateral arterial phase angiography of the left vertebral artery showing patent vasculature and the complete occlusion of the aneurysm.

Coil embolization was completed with bare platinum coils (Figure 4A, 4B). We used seven coils (MicroVention-Terumo, Inc., Tustin, CA) as follows: MicroPlex-18 12 × 43 Cosmos coil, MicroPlex-18 10 × 36 Cosmos coil, MicroPlex-10 15 × 40 VFC coil, MicroPlex-6 10 × 20 VFC coil, MicroPlex-10 8 × 37 Cosmos coil, MicroPlex-10 7 × 31 Cosmos coil, and a MicroPlex-10 6 × 26 Cosmos coil.

A follow-up angiogram obtained through the guiding catheter in the left vertebral artery demonstrated adequate occlusion of the aneurysm, and the LVIS device remained patent (Figure 4C, 4D).

The XperCT (Allura Xper FD20, Philips Medical Systems, Netherlands) scan following the endovascular procedure did not show hemorrhage. The XperCT, using an intracranial stent protocol, demonstrated the LVIS device was well-expanded against the vessel wall and was across the neck of the large aneurysm.

Hospital course

The patient was extubated in the Neuro Interventional Suite and was admitted to the Neuro Intensive Care Unit for close monitoring. Her neurological examinations were nonfocal. The dual antiplatelets (clopidogrel, 75 mg daily, and aspirin, 325 mg daily) were continued and a short course of dexamethasone, 4 mg every six hours was started to minimize the inflammatory response induced by the aneurysm thrombosis. On the second day after the endovascular occlusion of the aneurysm, the patient developed hallucinations and displayed agitation, which was attributed to steroid-induced psychosis. Her brain images were unremarkable, and the dexamethasone was discontinued. She remained clinically stable and was discharged home without encountering complications.

## Discussion

Vertebrobasilar junction aneurysms are very rare [1-3]. Their incidence is approximately 0.33% of all intracranial aneurysms [4-5]. Fenestration of the basilar artery is a recognized vascular variation with dual endothelium-lined vascular lumens which is usually due to a failure of fusion of the paired longitudinal neural arteries during the fifth week of embryonic life [4-5]. The histological abnormalities, namely, the elastin defect and lack of media within the fenestration along with the alteration in the blood flow hemodynamics, are responsible for the development of an aneurysm at the vertebrobasilar fenestrated junction [6-9].

The management of these fenestrated aneurysms is extremely difficult and challenging. The location of these aneurysms is proximal to the brainstem, and the wide varieties of the fenestrations complex and presence of perforator vessels make the microsurgical approach almost impossible [1-5]. An endovascular-based approach is becoming the first-line treatment, and meticulous preprocedural planning is extremely important to study the exact anatomy of the aneurysm-fenestration complex to determine the most appropriate endovascular therapeutic technique [10]. Few cases have been reported with successful outcomes using different endovascular modalities, including coiling, stent or balloon-assisted coiling, liquid embolic agents, and flow diversion devices [1-10].

This case was challenging in many ways. Our patient was morbidly obese, and her body habitus made optimal angiographic exposure of the aneurysm difficult, so we had to extend the neck of the patient for better exposure. The right vertebral artery was hypoplastic and ended in a PICA. Furthermore, the neck of the aneurysm was wide, fusiform, and involved both lumens of the fenestration with one dominant limb (i.e., the left branch of the fenestration), so the proposed use of a flow diversion device was not feasible. We decided to reconstruct the vasculature using one stent to support the coiling of the aneurysmal sac with preserved flow proximally, distally, and within the dominant limb of the fenestration.

## Conclusions

Vertebrobasilar junction aneurysms are very rare. A microsurgical approach is difficult because of the inherently complex anatomy of these vascular lesions. Endovascular therapy should be considered the first-line approach. Accurate preprocedural planning is critical to choosing the best endovascular technique and allowing for the best potential outcomes for the patient.
